# Intestinal Stem Cells and Immune Cell Relationships: Potential Therapeutic Targets for Inflammatory Bowel Diseases

**DOI:** 10.3389/fimmu.2020.623691

**Published:** 2021-01-20

**Authors:** Qihang Hou, Jingxi Huang, Hammed Ayansola, Hori Masatoshi, Bingkun Zhang

**Affiliations:** ^1^State Key Laboratory of Animal Nutrition, Department of Animal Nutrition & Feed Science, College of Animal Science & Technology, China Agricultural University, Haidian District, Beijing, China; ^2^Department of Veterinary Pharmacology, Graduate School of Agricultural and Life Sciences, The University of Tokyo, Bunkyo-ku, Tokyo, Japan

**Keywords:** intestinal stem cell, immune cell, inflammatory bowel disease, cytokine, organoids

## Abstract

The mammalian intestine is the largest immune organ that contains the intestinal stem cells (ISC), differentiated epithelial cells (enterocytes, Paneth cells, goblet cells, tuft cells, etc.), and gut resident-immune cells (T cells, B cells, dendritic cells, innate lymphoid cell, etc.). Inflammatory bowel disease (IBD), a chronic inflammatory disease characterized by mucosa damage and inflammation, threatens the integrity of the intestine. The continuous renewal and repair of intestinal mucosal epithelium after injury depend on ISCs. Inflamed mucosa healing could be a new target for the improvement of clinical symptoms, disease recurrence, and resection-free survival in IBD treated patients. The knowledge about the connections between ISC and immune cells is expanding with the development of *in vitro* intestinal organoid culture and single-cell RNA sequencing technology. Recent findings implicate that immune cells such as T cells, ILCs, dendritic cells, and macrophages and cytokines secreted by these cells are critical in the regeneration of ISCs and intestinal epithelium. Transplantation of ISC to the inflamed mucosa may be a new therapeutic approach to reconstruct the epithelial barrier in IBD. Considering the links between ISC and immune cells, we predict that the integration of biological agents and ISC transplantation will revolutionize the future therapy of IBD patients.

## Introduction

The mammalian intestinal tract is the main organ for nutrient digestion and absorption in the body. Moreover, it serves as the first barrier mechanism of the body defense system. In fact, intestinal health is closely related to the host’s health status, nutrition, environment, psychological state, and intestinal flora composition (1, 2). Anatomically, the gut is divided into two parts: the small intestine and colon. As the largest immune organ in the body, the mammalian intestine is known to be exposed to different antigens from commensal bacteria, diets, and pathogens. There are many immune cells that differ in frequency and location throughout the whole intestine (3). As a result, the intestinal epithelium mainly hosts T cells; whereas, the lamina propria contains both the innate and adaptive immune cells, including B cells, T cells, ILCs, dendritic cells, macrophages, eosinophils, and mast cells (3). Both innate and adaptive immunities are linked to maintaining intestinal homeostasis. Therefore, any perturbation in the intestinal homeostasis could potentially induce multiple intestinal diseases such as IBD, infectious diseases, diarrhea, and cancer in severe conditions (4).

**Figure 1:**
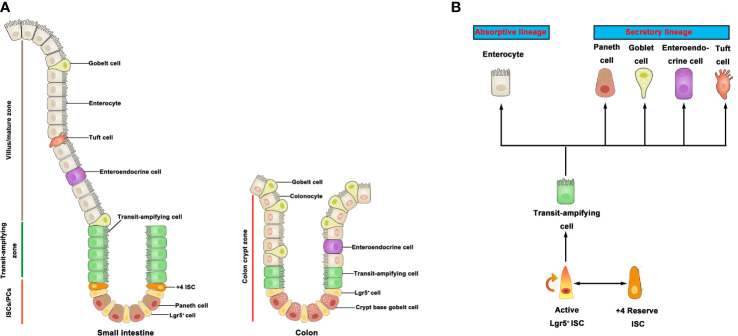
Intestinal stem cells and differentiated progeny. **(A)** The intestinal epithelium is covered with a monolayer of epithelial cells. ISC generate TA cells in the crypts. TA cells migrate upward along the villi-crypt axis and differentiate into various epithelial cells. ISC are located at the bottom of the intestinal crypts. **(B)** Lgr5^+^ ISC generate TA cells, which can produce two mature epithelial cell lineages: absorptive type (enterocytes) and secretory type (goblet cells, Paneth cells, tuft cells, and enteroendocrines). +4 ISC have been proposed as a quiescent stem cell population. ISC, intestinal stem cell; TA, transit-amplifying.

In mammals, the intestinal epithelium is the most active self-regenerative tissue (5) and constantly renewed by ISC in the crypt bottom (6). ISC is capable of differentiating into progenitor cells, and these newly formed cells proliferate and differentiate along the crypt-villus axis of the small intestine and colon (**Figure 1A**). Leucine-rich repeat-containing G-protein coupled receptor 5 (Lgr5) is an important marker of active ISC identified to generate differentiated epithelium cell types over a long period of time (7). In addition, another population of quiescent “reserve” ISCs is located at the so-called ‘+4’ position (8), whereby Bmi1, mTert, Hopx, and Lrig1 have been identified as markers of +4 ISCs (9–12). Studies have shown that the Lgr5^+^ ISC is a highly active ISC necessary for intestinal epithelium renewal (7). On the contrary, the +4 ISCs are activated following injury, then regenerate Lgr5^+^ ISC to replenish the stem cell pool (8). Moreover, active stem cells would possibly have the capacity to replace lost or damaged quiescent stem cells in certain conditions (13). There is a balance among rapid-cycling, easy-to-damage ISC, and quiescent +4 ISC to sustain self-renewal and protect against flexible damage in the intestinal crypt. In the small intestine, ISC can differentiate into five major cell types but only differentiate into three major cell types in the colon. Although the primary epithelial cells are known to be absorptive enterocytes in the small intestine, the intestinal epithelium also contains some secretory cell lineage, including Paneth cells that support the ISC niche and secrete antimicrobial peptides, mucus-producing goblet cells, various hormone-secreting enteroendocrine cells, and M cells and tuft cells (6) (**Figure 1B**). It is worth noting that colonic crypts lack Paneth cells, but there are Paneth-like cells called crypt base goblet cells (14, 15). Currently, the gene characteristics of crypt base goblet cells are hypothesized to be between Paneth cell and goblet cell; however, their functional significance is still unclear. The ISC niche consists of both a mesenchymal component and an epithelial component (Paneth cells). The mesenchymal compartment contains multiple stromal cell populations, such as fibroblasts, myofibroblasts, and smooth muscle cells, which secrete multiple growth factors for the maintenance of ISC function (16). Paneth cells, on the other hand, adjoin ISC and provide essential niche signals such as Delta-like 1/4, EGF, and Wnts to support ISC homeostasis in small intestine (17, 18). In line with the available data, many reviews have discussed the role of mesenchymal cells and Paneth cells in the ISC niche (5, 19–21). In recent years, however, new data are now showing that the interaction between resident immune cells and ISC is crucial for the regenerative capacity of the intestinal epithelium cells. Emerging insights from microbiome research reveal that gut microbiota-derived signals and molecules influence the development/activity of ISC. A recent long review surveyed the literature on gut microbiota-host crosstalk, highlighting the effects of gut microbial metabolites on intestinal stem cells (22).

IBD, one of the most common intestinal diseases, comprises both Crohn’s disease (CD) and ulcerative colitis (UC), which are the chronic and immunologically inflammatory disorders in the gastrointestinal tract (23). Typical clinical symptoms of IBDs include diarrhea, abdominal pain, and rectal bleeding (24), arising from immunological dissonance, microbiota disorder, and epithelial barrier dysfunction in the intestine (25). IBDs are prevalent globally, especially in western countries (26). To date, the pathogeny and pathology of IBD are not lucid; however, interactions among genetic factors, environmental factors, microbiome, and the immune system are considered to play a key role in the nosogenesis of IBD (27, 28). Moreover, IBD is mainly induced by immune dysfunction in the intestinal immune system. Specifically, mucosal immune cells, such as T cells, macrophages, dendritic cells, and ILCs, regulate the intestinal homeostasis by secreting multiply cytokines (29). These cytokines are directly linked to IBD pathogenesis and known to control intestinal inflammation associated with IBD (29, 30). The microbiota provides crucial signals for the development and function of the immune system. The gut microbiota and their metabolites are not only necessary for immune homeostasis, they also influence the susceptibility of the host to many immune-mediated diseases and disorders (31). Over the last 2 decades, a large number of studies have shown that gut microbiota and their metabolites play a key role in the pathogenesis of IBD (32). However, the gut microbiota-immunity-IBD axis is extremely complicated. The interaction between gut microbiota and host immunity and these nets in IBD pathogenesis have been discussed and summarized in many authoritative reviews.

The development of *in vitro* intestinal organoid culture and single-cell RNA sequencing technology proffer improved techniques to better understand the interaction between ISC and immune cells (33–35). For example, activated ILC3s produce IL-22, which acts on ISC to induce intestinal epithelium regeneration through STAT3 signals (35). A recent study also demonstrates that ISC fate is modulated by interactions between ISC and T cells. IL-10 produced by regulatory T (Treg) cells increased the ISC numbers, while IL-13 and IL-17 produced by T helper (TH) cells resulted in the expansion of differentiated cells and depletion of the ISC pool (33). Hence, this review paper addressed the regulation of immune cells such as T cells, ILCs, dendritic cells, and macrophages on ISC fate and function within the scope of future therapeutic approaches in IBD.

## Intestinal Stem Cell Niche

The intestinal epithelium constantly renews by sequential proliferation and differentiation (5), from ISC to progenitor cells, to special types of epithelial cells for the purpose of maintaining gut homeostasis (5, 7). The ISC niche provides the microenvironment required to maintain ISC proliferation and differentiation. In this niche, multiple cells provide cellular signals that promote ISC function and also ensure that they have sufficient turnover to differentiate into a normal epithelial barrier against the development of tumor cells. Currently, the use of intestinal organoids helps to advance our understanding the composition of the ISC niche environment (36). From studies, both stromal cells and Paneth cells supply complex paracrine signals, including Wnt, Notch, BMP, and Hedgehog, that mediate the interactions between ISC proliferation and differentiation (37). Additional data have proven the implications of immune cells in gut homeostasis (36). Communication between immune cells and intestinal epithelial cells serves as the key mediators that preserve the integrity of the gut system (2, 38, 39). For instance, tissue-resident immune cells are involved in the regeneration of the intestinal cells (2, 33, 40). Altogether, further advancements in the *in vitro* ISC-immune cell co-culture system will clarify the complex mechanisms, through which the intrinsic factors of immune cells regulate ISC fate.

### Intestinal Mesenchymal Cells

Intestinal mesenchymal cells, such as fibroblasts, myofibroblast, endothelial cells, and smooth muscle cells, have provided both structural support as well as factors such as Wnt ligands and BMP antagonists that regulate ISC activities. The depletion of Foxl1^+^ mesenchymal cells in a recent experiment caused ISC dysfunction due to decreased WNT signals that eventually led to intestinal failure in mice (41). In a similar study, Gli1^+^ mesenchymal cells are the source of WNT2B and are essential for the function of ISC (42). A significant characterization of the ISC niche is the origins of various types of mesenchymal cells (like WNT2B, R-spondin 1, Gremlin 1, and CD34 +). The aforementioned studies established the relevance of the mesenchyme cells for supplying WNT ligands, BMP inhibitors, and R-spondins.

Besides that, there is a bidirectional relationship between epithelial-mesenchyme. The intestinal epithelium stimulates the hedgehog signaling pathway through the activation of the ligands in the surrounding mesenchyme as a mechanism to promote the growth of both the mesenchyme and smooth muscle cells during development in the adult organism (43). Likewise, other intestinal mesenchymal cells, such as interstitial cells of Cajal and PDGFRα-positive fibroblast-like cells, exist in the muscle layer of the gut (44). The two cells and the intestinal plexus form a network so as to warp the digestive tract as well as many resident macrophages that fix on the same site. Overall, there is a call to further investigate the humoral factors that underlie the connections between these stromal cell groups in the gastrointestinal muscle layer and ISC in the mucosal layer.

### Paneth Cells

The development of ISC-derived intestinal organoid culture proved the ISC niche could function independently in the absence of mesenchymal cells (45). In the small intestinal crypt, Paneth cells are adjacent to ISC and provide the necessary niche signals in their environment (18). Antimicrobial peptides secreted by Paneth cells, for example, are integral to the defense of the ISC niche (46, 47). Moreover, Paneth cells express multiply signaling molecules such as WNTs and the Notch ligands, which are essential for the maintenance of the ISC niche (17). The functions of these signals are explicit; however, the importance of the Paneth cells in the ISC niche is controversial. Atoh1 (also known as Math1, Hath1 in mouse and human respectively) is considered a part of the Notch signaling pathway and regulates Notch-based ISC fate decisions (48). Previous studies have reported that the ablation of Paneth cells in *Atoh1^-/-^* mice did not affect ISC proliferation (49, 50). The findings argued that the presence of EGF and WNT could mediate the Notch signaling as alternatives to Paneth cells. Unfortunately, *Sox9* or *Gfli1* gene knockout in mice depletes Paneth cells but results in the loss of ISC (51–53). In a similar study, the diphtheria-toxin receptor gene is knocked into the murine Reg4 locus to eliminate crypt base goblet cells. In the large intestine, Reg4^+^ crypt base goblet cell acts as the marker of the Paneth cell functions. Ablation of crypt base goblet cells not only results in the loss of ISC from colonic crypts but also disrupts colon homeostasis and organoid growth (54). Nevertheless, some researchers opine that gene-knockout mice might not be an ideal model to justify whether Paneth cells are key to the ISC niche. On this notion, there is still the need to elucidate whether Paneth cells are integral to the maintenance of ISC niche homeostasis in future studies.

### Immune Cells

**Figure 2:**
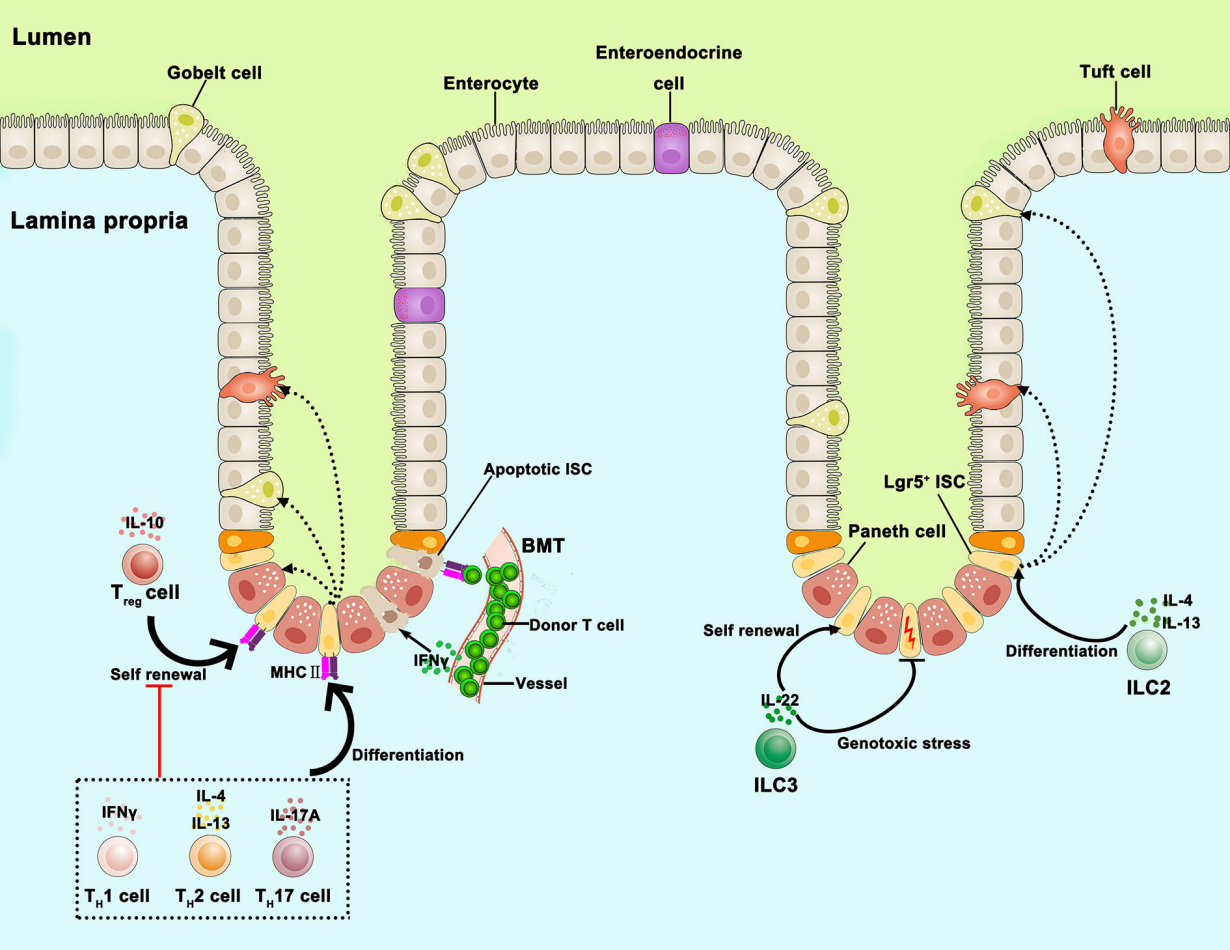
The link between the intestinal stem cells and immune cells. The role of immune cells such as T cells and ILCs, as well as cytokines secreted by these immune cells on the regulation of ISC function. T_reg_ cells (IL-10) promote ISC renewal, while T_H_1 (IFNγ), T_H_2 (IL-4, IL-13), and T_H_17 cells (IL-17A) suppress ISC renewal and promote differentiation; T_H_1(IFNγ) and T_H_2 (IL-4, IL-13) cells promote specific differentiation toward Paneth cells and tuft cells, respectively; donor T cells (IFNγ) act on ISC by triggering apoptosis in GVHD; MHCII is important for interactions between ISC and T cells. ILC2s (IL-4, IL-13) promote specific differentiation toward goblet cells and tuft cells; ILC3s (IL-22) promote ISC proliferation through STAT3 signals; protect ISC against genotoxic stress. IL, interleukin; IFNγ, interferon γ; ISC, intestinal stem cell; ILC, innate lymphoid cell; T_H_, T helper; T_reg_ cells, regulatory T cells; GVHD, graft versus host disease, MHCII, major histocompatibility complex II; STAT3, signal transducer, and activator of transcription 3.

The knowledge about the connections between ISC and immune cells is expanding with the development of *in vitro* intestinal organoid culture and single-cell RNA sequencing technology. Recent findings implicate immune cells in the regeneration of the gut (2, 33, 40). Cytokines secreted from immune cells also participate in ISC regulation (33–35). Thus, this section will review the functions of immune cells such as T cells, ILCs, dendritic cells, and macrophages, as well as cytokines secreted by these cells in the ISC niche (**Figure 2**) (**Table 1**).

**Table 1:** The link between the intestinal stem cells and immune cells.

Immune cell type	Cytokines	Research models	Function in ISC	Refs
**T_H_1 cells**	IFNγ	T_H_1 cells/IFNγ-IOs co-culture models	Suppress ISC renewal and promote specific differentiation toward Paneth cells	(33)
**T_H_2 cells**	IL-4, IL-13	T_H_2 cells/IL-4, IL-13-IOs co-culture models	Suppress ISC renewal and promote specific differentiation toward tuft cells	(33)
**T_H_17 cells**	IL-17A	T_H_17 cells/IL-17A-IOs co-culture models	Reduce ISC renewal and promote differentiation	(33)
**T_reg_ cells**	IL-10	T_reg_ cell/IL-10-IOs co-culture models	Promote ISC renewal	(33)
**T cells**	IFNγ	GVHD model *in vivo* and T cell-IOs co-culture model *in vitro*	Act on ISC by triggering apoptosis	(55)
**Fetal CD4^+^ Tem cells**	TNF-α	Fetal Tem cells-IOs co-culture model	Low doses of TNF-α promote the proliferation of fetal ISC, high levels of TNF-α induce fetal ISC apoptosis and reduce ISC populations	(56)
**ILC2s**	IL-4, IL-13	Activated ILC2s-IOs co-culture model	Promote specific differentiation toward goblet cells and tuft cells	(57–59)
**ILC3s**	IL-22	Activated ILC3s/IL-22-IOs co-culture models	Promote ISC proliferation through STAT3 signals; protects ISC against genotoxic stress	(34, 35, 60)
**Jurkat T cells**	IL-2	Jurkat T cells/IL-2-hIOs co-culture models	Induce the maturation of hIOs through STAT3 signals	(61)
**Macrophages**	IL-6, IL-8, IFNγ, and TGFβ1	Macrophages-IOs co-culture models	Maintain the ISC niche in the small intestine; enhance the maturation of the intestinal epithelium, and thickening the physical barrier	(62, 63)
**DCs**	IL-1β, IL-6, IL-15, and IL-17A	Bone marrow-derived DCs-IOs co-culture models	NF-κB2 signaling in organoids modulates enterocyte responses to secreted factors from bone marrow-derived DCs	(64)

IL, interleukin; IFNγ, interferon γ; ISC, intestinal stem cells; IOs, intestinal organoids; ILCs, innate lymphoid cells; T_H_, T helper; T_reg_ cells, regulatory T cells; DCs, dendritic cells; GVHD, graft versus host disease; STAT3, signal transducer and activator of transcription 3; TNF, tumor necrosis factor; Tem, T effector memory; hIOs, Human pluripotent stem cell-derived intestinal organoids; STAT3, signal transducer and activator of transcription 3.

#### Immune Cell-Organoid Co-Culture Model Systems

**Figure 3:**
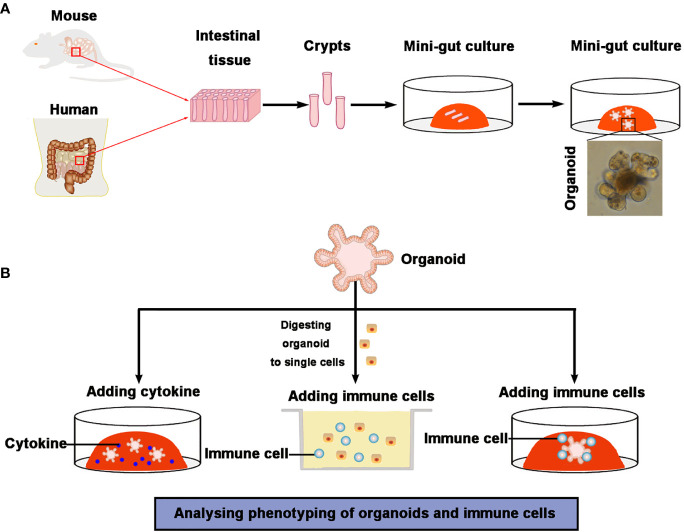
Intestinal organoid–immune cell co-culture models. **(A)** Crypts are isolated from the intestinal tissue and then embedded into Matrigel with culture medium. Intestinal organoids are formed by crypts. **(B)** Several intestinal organoids and immune cells and/or cytokines co-culture systems have been established. Treatment of organoids with cytokines is used to estimate the effect of immune cell-derived cytokines on intestinal epithelial cells (especially ISC). Organoids are digested to single cells and then co-cultured with immune cells, which is used to assess the interaction of immune cells and epithelial cells. The addition of (activated) immune cells, such as innate lymphoid cells or T cells, to complete organoids is used to estimate the interactions between epithelial cells and immune cells.

The use of experimental animal models has enhanced our understandings of the intricacies of many biological processes. Unfortunately, the complex nature of the physiology of these models renders it challenging to study the mechanisms that underlie the interactions between intestinal epithelial cells and immune cells. As a result, intestinal organoid culture system (45, 65) *in vivo* models are designed to investigate this research gap because they are applicable to disease modeling, drug development, and *in vitro (*66) studies of cellular differentiation (**Figure 3A**). ISC-derived organoids contain all types of differentiated epithelial cells that allow for proliferation and differentiation of intestinal epithelium under defined conditions. Co-culture models of intestinal organoids with different immune cell types enable *in vitro* studies of these complicated interaction networks (**Figure 3B**).

#### T Cells

T cells found in the gastrointestinal tract, on the one hand, are contributing to the immunity of the intestine, and on the other hand, are promising targets for immune-mediated intestinal damage therapy. Recent studies demonstrated that both CD4^+^ and CD8^+^ T cells had the potential to mediate injury to the ISC compartment in graft-versus-host disease model. The disease-causing T cells, after bone marrow transplantation (BMT)-mediated GI damage, targets the ISC niche as the primary site in the intestine under three-dimensional imaging examination (67). In addition, the recruitment of disease-causing T cells to the crypt base region resulted in the loss of ISC that expresses both the major histocompatibility complex class (MHC) I and II (67). In a similar report, another study explored the changes in the ISC niche using T cell-mediated injury in graft-versus-host disease (GVHD) model *in vivo* and intestinal organoid-T cell co-culture *in vitro (*55). This study showed that both ISC and Paneth cell numbers per crypt were markedly decreased in mice that received bone marrows or allogeneic T cells. In comparison, these mice showed a notable increase in the density of both T cells associated with lamina propria and intraepithelial in the crypt region (55).

Recently, scientists have investigated the co-culture of human and murine intestinal organoid-T cells to understand the molecular mechanisms that potentially cause the loss of ISC in disease models. Murine alloreactive T cells significantly reduced intestinal organoid numbers, whereas human allogeneic CD8^+^ cytotoxic T cells inhibited the efficiency of intestinal organoids. Moreover, CD4^+^ T cell-derived IFNγ directly induced ISC apoptosis through JAK/STAT signaling in the absence of Paneth cells (55). Altogether, these data broaden our knowledge on the interaction between immune cells and ISC, thus presents ISC as direct targets of IFNγ produced by T cells in immune-mediated gut damage. In the future, the inhibition of JAK/STAT signaling could be a new therapeutic approach to ameliorate GVHD-induced gut damage when the effects of T cell-derived IFNγ on ISC are taken into account.

Meanwhile, a recent study reported that organoids derived from fetal intestine were utilized to explore the interactions between the epithelial cell and immune cell in fetal development (68). Single-cell RNA sequencing in a recent report identified a population of CD4^+^ effector memory T cells (56). These Tem cells could secrete tumor necrosis factor α (TNF-α) (56). In line with this idea, Schreurs et al. used human fetal intestinal organoids-CD4^+^ Tem cells co-culture model to examine the role of TNF-α on epithelial cells. Their results revealed that through TNF-a mediation, low numbers of fetal intestinal CD4^+^ Tem cells enhanced the proliferation of ISC, while on the contrary, high numbers of fetal intestinal CD4^+^ Tem cells impaired ISC function (56). The detrimental effects of a high dose of TNF-a on ISC development were consistent with observations in infants suffering from necrotizing enterocolitis (56).

The interaction between ISC and T helper (T_H_) cells contributes to ISC function and intestinal epithelial remodeling, as reported in a recent publication (33). This study utilized single-cell RNA sequencing to identify the subsets of Lgr5^+^ ISC that could highly express MHCII molecule. *In vitro* studies with intestinal organoids demonstrated that ISC expressing MHCII has the greatest antigen-presenting ability in intestinal epithelial cells. Moreover, MHCII mediated the communication between the T_H_ cells and these ISC, which led to T_H_ cells activation in an antigen-presenting manner (33). Intestinal organoids were co-cultured with various T_H_ cell subsets or specific marker cytokines to observe effects on ISC in order to further elucidate ISC and T helper (T_H_) cells interactions. Supplementation with pre-activated T_H_2 cells or IL-14 and IL-13 resulted in the depletion of the ISC pool. Similarly, co-culture with pre-activated T_H_1 and T_H_17 cells, or cytokines secreted by these cells, such as IFNγ and IL-17, led to the decrease in the number of ISC but increase the number of transit-amplifying cells. On the contrary, co-culture with regulatory T (T_reg_) cells or IL-10, however, led to the expansion of the ISC pool (33). In another report, ISC co-culture with human T cells resulted in the *in vitro* maturation of human pluripotent stem cell-derived intestinal organoids (hIOs), which identified IL-2 as the key factor that induced the maturation *via* signal transducer and activator of transcription 3 (STAT3) signaling (61). Their additional data also demonstrated that co-culture with IL-2 could increase the expression levels of mature intestinal markers as well as the intestine-specific functional activities in hIOs (61).

The deduction from the above studies suggests that ISC and T cell interactions directly participated in the regulation of ISC fate. ISC, in particular, is capable of sensing T cells *via* MHCII interactions, which could lead to T cells activation. Therefore, more studies are required to further clarify the nexus between intestinal immunity and intestinal epithelial barrier function.

#### Innate Lymphoid Cells

Innate lymphoid cells (ILCs) act as critical regulators of intestinal mucosal immunology and are crucial for maintaining gut homeostasis and epithelium barrier integrity (69). ILCs are divided into three major groups according to the cytokines they produce, their phenotype, and their developmental pathways such as ILC1s, ILC2s, and ILC3s (70). Several studies have associated ILCs-secreted cytokines with the fate of ISC activities. Specifically, IL-22 was found to regulate the maintenance and differentiation of ISC (35, 60, 71, 72) as well as its protection against DNA damage (34). Many studies have identified that IL-22 plays a critical role in the repair of the intestinal epithelium during DSS-induced colitis (73–75). To date, the mechanisms of IL-22 involvement in the regulation of ISC fate are still poorly understood; however, it is known that ISC could express IL-22 receptor (7, 60). IL-22 was associated with the intestinal epithelial repair of GVHD and BMT, where allogeneic T cells inhibit ISC functions (35, 60). Mechanistically, IL-22 could activate the STAT3 signaling pathway in Lgr5^+^ ISC and promote the maintenance and differentiation of ISC without relying on Paneth cells (60). IL-22 improved not only the survival of ISC but also its proliferation after irradiation, which is essential to the regeneration of damaged epithelium (35). This knowledge suggests that the IL-22-STAT3 axis might be a potential target for the therapy of IBD, and in addition, as a strategy to protect against the side effects of therapies from high intestinal toxicity, such as BMT and GVHD. However, one latest study challenged the perceptions of IL-22 as a beneficial cytokine in IBD (76). The study described that in colonic epithelial cells, IL-22 induced endoplasmic reticulum (ER) stress response and transcriptional program. In CD patients, IL-22-responsive transcriptional modules and ER stress response modules are highly expressed in the colon. Blocking IL-22 ameliorated colonic epithelial ER stress and attenuated the IL-22-dependent model of chronic colitis in mice (76). This evidence thus offered new insights that IL-22 acts as a double-edged sword in chronic intestinal inflammation.

Environmental genotoxic factors can also induce mutations of ISC, which contribute to cancer development and malignant transformation (77–79). The DNA damage response (DDR) acts as an evolutionarily conserved response pathway at the cellular level to maintain genome integrity in ISC (80). A recent publication showed that IL-22, mainly produced by ILC3s and γδ T cells in the intestinal mucosa, is a critical conditioner of the DDR pathway in colon stem cells (CSCs) (34). IL-22 effectively activated the DDR in CSCs by expressing the IL-22 receptor after DNA damage. When IL-22 signals were deprived, ISC exposed to carcinogens exhibited significant mutations that could likely lead to the development of colon cancer (34). In addition, some metabolites of glucosinolates were the primary source of genotoxic stress in the intestinal epithelium, which act as ligands of the aryl hydrocarbon receptor (AhR) to mediate the production of IL-22 in ILC3s and γδ T cells through AhR-mediated signaling (34). Moreover, in our previous study, we established intestinal organoids and lamina propria lymphocytes (LPLs) co-cultured system to explore the protective effect of *Lactobacillus reuteri* on integrity of intestinal mucosa. We demonstrated that *Lactobacillus reuteri* metabolite indole-3-aldehyde stimulated LPLs to secret IL-22 and promoted ISC-mediated intestinal epithelial regeneration (72).

Group 2 innate lymphoid cells (ILC2s) also affect ISC fate. For instance, IL-13 produced by ILC2 has been demonstrated to accelerate the differentiation of goblet cells in the intestinal organoid model. In addition, IL-33 secreted by intestinal epithelium was shown to promote IL-13 production by ILC2s (81). Furthermore, several publications have shown that the differentiation of tuft cells, which is responsible for type II immune responses, was regulated by IL-4 and IL-13 in intestine (57–59). It is quite interesting to investigate the links between the intestinal immune system and epithelium mediated by tuft cells. For example, tuft cells function as immune sentinels that respond to the presence of parasites in the intestine. Additionally, succinate derived from helminth induced tuft cells to secrete IL-25 (59, 82). Furthermore, IL-25 recruits ILC2s in intestinal mucosa thereafter stimulates these cells to secrete IL-4 and IL-13, resulting in the removal of parasitic worms through increased mucus production from hyperplastic goblet cell (59). Even as these studies demonstrated that IL-4 and IL-13 produced by ILC2s generated more differentiated epithelial cells such as goblet and tuft cells in the intestine, there is still the need to explore further direct interaction between ILC2s and ISC as well as underlying molecular mechanisms.

#### Other Immune Cells

Macrophages and dendritic cells exist throughout the lamina propria of the gut and contribute to innate and adaptive immunity, thus maintaining gut homeostasis. The co-culture of intestinal organoids with macrophages and dendritic cells has also been successfully performed to study complex networks between immune cells and ISC (62–64). Macrophages have been shown to promote mucosal repair through activation of Wnt-signaling in a mouse model of IBD (83). A recent study demonstrated that colony-stimulating factor 1 (CSF1)-dependent macrophages in the gut wall are essential to maintain the ISC niche in the small intestine (62). It also suggested that CSF1 treatment has the potential to restore the intestinal epithelial barrier following damage caused by inflammation and chemotherapy. In another study, a human enteroid-macrophage co-culture model was built to investigate host gut-pathogen interactions (63). In this study, cytokines secreted by macrophages such as IL-6, IL-8, IFNγ, and TGFβ1 might contribute to the potential roles of macrophages in enhancing the maturation of the intestinal epithelium and the thickening of the physical barrier (63). Similarly, Jones et al. demonstrated that cytokines released by bone marrow-derived dendritic cells could modulate intestinal barrier integrity, intestinal cell proliferation, and cell death through NF-κB2 signaling (64). Despite the fact that these studies expand our knowledge about the immune cell-ISC interactions, we must realize that these networks may be far more complicated than we currently understand.

## Cytokine-Based Immune Therapy in Inflammatory Bowel Disease

As a chronic inflammatory disease, IBD is mainly induced by immune dysfunction in the intestinal immune system. Specifically, intestinal immune cells, such as T cells, macrophages, dendritic cells, and ILCs, regulate the intestinal homeostasis *via* producing multiply cytokines (29). In previous studies, cytokines have direct links with the pathogenesis of IBD and are implicated in the modulation of intestinal inflammation and clinical symptoms of IBD (29, 30) (**Table 2**). IL-2^-/-^ and IL-10^-/-^ mice developed spontaneous colitis, which highlighted the critical roles of cytokines in IBD (30). In addition, studies in the 1990s demonstrated that the administration of anti-inflammatory cytokines could prevent IBD (110), and IL-12 (a pro-inflammatory cytokine) antibody could be used for the therapy of colitis (117) in mouse models. In the following decades, some supporting studies revealed that intestinal immune cells (such as mucosal effector T cells, T_reg_ cells, ILCs, and dendritic cells) could produce multiple pro-inflammatory and anti-inflammatory cytokines in response to environmental factors in patients with IBD and mouse models of colitis. Moreover, the development of mucosal inflammation is regulated by the balance between pro-inflammatory and anti-inflammatory cytokines in the intestinal mucosa of patients with IBD (29). As a result, scientists have made great efforts in recent years to explore the efficacy of anti-inflammatory cytokines and neutralizing antibodies for pro-inflammatory cytokines in the clinical therapy of IBD.

**Table 2:** Selected key cytokines in inflammatory bowel disease.

Cytokine	The source in the intestine	Disease	Potential function in IBD	Therapeutic targets in IBD
**IL-1**	Macrophages and neutrophils	UC, CD	Enhances IL-6 production by macrophages, stimulates ILCs and promotes tumor development	Anti-IL1Ra ameliorates DSS colitis (84);IL1-RA (case study) (85, 86)
**IL-6**	Macrophages, fibroblasts, and T cells	CD	Activates T cells, inhibits apoptosis, accelerates proliferation of epithelial cells, and supports tumor growth	Anti-IL6R ameliorates TNBS, Il10^-/-^ and T cell transfer colitis (87, 88); anti-IL6R and anti-IL6 (clinical improvement) (89, 90)
**IL-10**	T cells	CD	Inhibits pro-inflammatory cytokine production by APCs and T cells, induces STAT3 signals in Treg cells	IL-10-producing L. lactis ameliorates Il10^-/-^ and DSS colitis (91); recombinant IL-10 (no effect) (92) and IL-10-producing L. lactis (safe) (93)
**IL-13**	T cells and iNKT cells	UC	Regulates intestinal barrier functions	Anti-IL-13 suppresses disease in an oxazolone-induced model of colitis (94); Anrukinzumab and tralokinumab (efficacy currently unknown) (95)
**IL-12, IL-23**	Macrophages and DCs	CD	Activates mucosal immune cells such as T cells and ILCs, enhances cytokine production such as TNF-α	Anti-IL-12p40 and anti-IL-23p19 ameliorate Helicobacter hepaticus-induced innate colitis and Il10^-/-^ colitis (96–98); anti-IL-12p40 and anti-IL-23p19 (clinical improvement in CD and UC) (99–101)
**IL-17A**	T cells and ILCs	CD	Mediates pro-inflammatory and anti-inflammatory effects and induces pro-fibrotic functions	Anti-IL-17A aggravates DSS colitis (102) but ameliorates Il17f^-/-^ T cell transfer colitis (103); anti-IL-17A (104) and anti-IL- 17RA (105) (no effect)
**IL-18**	Macrophages, DC and epithelial cells	UC, CD	Enhances the production of pro-inflammatory cytokines in gut	Anti-IL-18 ameliorates DSS colitis (106); rhIL18BP (case study) (107)
**IL-22**	γδ and αβ T cells, ILCs and DCs	UC, CD	Induces production of antimicrobial peptides and proliferation of epithelial cells, favors tumor development *via* STAT3 activation	IL-22 application ameliorates TCRa^-/-^ and DSS colitis (108, 109)
**TNF**	Macrophages, DCs, and T cells	UC, CD	Induces pro-inflammatory cytokine production and angiogenesis, induces death of intestinal epithelial cells, mediates T cell resistance against apoptosis	Anti-TNF ameliorates T cell transfer colitis (110); anti-TNF (approved for UC and CD) (111–113)
**IFNα, IFNβ**	DCs	UC	Arguments intestinal epithelial regeneration and induce IL-10-producing cells	Recombinant IFNβ (no effect) (114, 115)
**IFNγ**	T cells and ILCs	CD	Activates macrophages, induces death of intestinal epithelial cells	Anti-IFNγ (no effect) (116)

IL, interleukin; IBD, inflammatory bowel disease; UC, ulcerative colitis; CD, Crohn’s disease; DCs, dendritic cells; ILCs, innate lymphoid cells; DSS, dextran sodium sulfate; Treg cells, regulatory T cells; APCs, antigen-presenting cells; STAT3, signal transducer and activator of transcription 3; TNF, tumor necrosis factor; IFN, interferon; rmIL18RB, recombination interleukin 18 receptor beta subunit.

The effects of many anti-inflammatory cytokines (such as IL-10, IL-11, and IFNβ) on the treatment of IBD have shown little promise so far (118–120). However, anti-TNF therapy (infliximab) poses to improve clinical symptoms and inflammation of the intestinal mucosa when administered to patients with CD (121). The introduction of drugs (such as golimumab, adalimumab, and certolizumab pegol) has positioned anti-TNF therapies as potent treatments for both UD and CD (27, 28, 122). Another pro-inflammatory cytokine, IL-6, is discovered to be participating in IBD pathogenesis. Circulating IL-6 and its agonistic IL-6-soluble receptor were increased in IBD patients (123). Previous studies revealed that the anti-IL-6 receptor antibody, tocilizumab, and IL-6 antibody, PF-04236921 yielded high clinical responses in Crohn’s disease patients (89, 124). On the contrary, neutralization of other pro-inflammatory cytokines such as IL-17A (with secukinumab) and IFNγ (with fontolizumab) had no beneficial effect on the treatment of CD (104, 125). In addition, only a subset of patients was able to record successful clinical outcomes after anti-cytokine therapies and cytokine signaling inhibitions (126–128). These studies suggest that the mucosal inflammation in patients with IBD is regulated by a complex network of cytokines in the intestinal mucosal community. These cytokine networks are vulnerable to intrinsic factors such as genetic, microbial, and immune systems during immune perturbation in IBD patients (29). Besides that, researchers should also consider the variation in the underlying mechanisms that could possibly induce intestinal inflammation among patients with IBD.

Multi-cytokine inhibitors that block multiple pro-inflammatory cytokine signals such as JAK-STAT signaling pathways could be used to improve the therapeutic effect in IBD. For example, JAK inhibitor (tofacitinib) has recently been demonstrated to be effective against UC but have no effect on CD in the initial clinical studies (128, 129). Therapeutics strategies that could be able to neutralize two or more pro-inflammatory cytokines have already been used e.g. Ustekinumab, which blocks IL-12/IL-23 (130). Clinical trials have shown that therapies targeting IL-23 are effective not only in CD but also in UC (99, 100). Another effective therapy could be personalized treatment for individual IBD patients as a way to enhance the therapeutic effect and minimize potential side effects. Nevertheless, detailed serum markers or cytokines levels of patients need to be tested in order to accomplish this goal. For example, a recent study used fluorescent molecular imaging of anti-TNF antibodies to predict CD response to biological therapy (131). However, more experimental validations are required before this technique can be put into clinical practice. Under this condition, new optimized ways for the delivery of targeted therapeutic drugs to the lesion region of the inflamed mucosa should be explored. In previous study trials, for instance, oral delivery of anti-TNF Nanobody and IL-27-producing lactobacilli attenuated experimentally induced colitis in mice (132, 133).

Over the last 3 decades, molecular mechanisms of cytokine biology in IBD have been explored mainly in experimental animal models and clinical studies, leading to their successful translation into drug targets. This provides a new possibility for the control of IBD symptoms and the long-term remission of IBD. Despite significant advances, cytokine therapies are only at the early stage, which renders it unsafe for human clinical trials. In other words, more in-depth and systematic researches tailored to specific disease conditions will be required for making informed deductions about cytokine dynamics and mechanisms of actions in order to better understand the potentials of cytokine therapies for clinical translations.

## Intestinal Stem Cell Therapy in Inflammatory Bowel Disease

New data showed that inflamed mucosa healing could be a new target for the improvement of clinical symptoms, disease recurrence, and resection-free survival in IBD treated patients (134–136). In line with this idea, TNF-α inhibitor (a biological agent) gave rise to mucosal healing from a portion of IBD patients, which signifies tremendous progress in IBD therapy (137). However, several patients did not respond to these biological agents—this challenge underlies the need to discover new approaches for achieving mucosal healing in IBD. For the case of patients that do not respond to biological agents, transplantation of ISC to the inflamed mucosa may be a new therapeutic approach to reconstruct the epithelial barrier in IBD. Over the past 2 decades, studies have indicated that mesenchymal stem cells (MSCs) and hematopoietic stem cells (HSCs) transplantation or transfusion have beneficial effects on IBD patients. A series of trials have been performed to demonstrate the effectiveness of MSCs transplantation in the treatment of the luminal and fistulizing type of CD. Allogeneic and autologous transfusion of MSCs could improve the symptoms of luminal CD patients with low risk of adverse events (138–143). Moreover, a latest clinical trial proved that allogeneic bone-marrow derived MSC therapy is effective and safe in luminal CD patients (144). Therefore, MSCs transplantation seems to be an effective and safe treatment for a portion of CD patients. In contrast, HSC transplantation (HSCT) cannot be recommended due to frequent serious adverse events (145), although some clinical trials using HSCT showed a certain effect on the treatment of IBD (146–149). Nevertheless, a latest retrospective study indicated that autologous HSCT is relatively safe and effective for refractory CD patients (150). Therefore, whether HSCT can be used as a good alternative therapy for refractory CD patients remains controversial.

**Figure 4:**
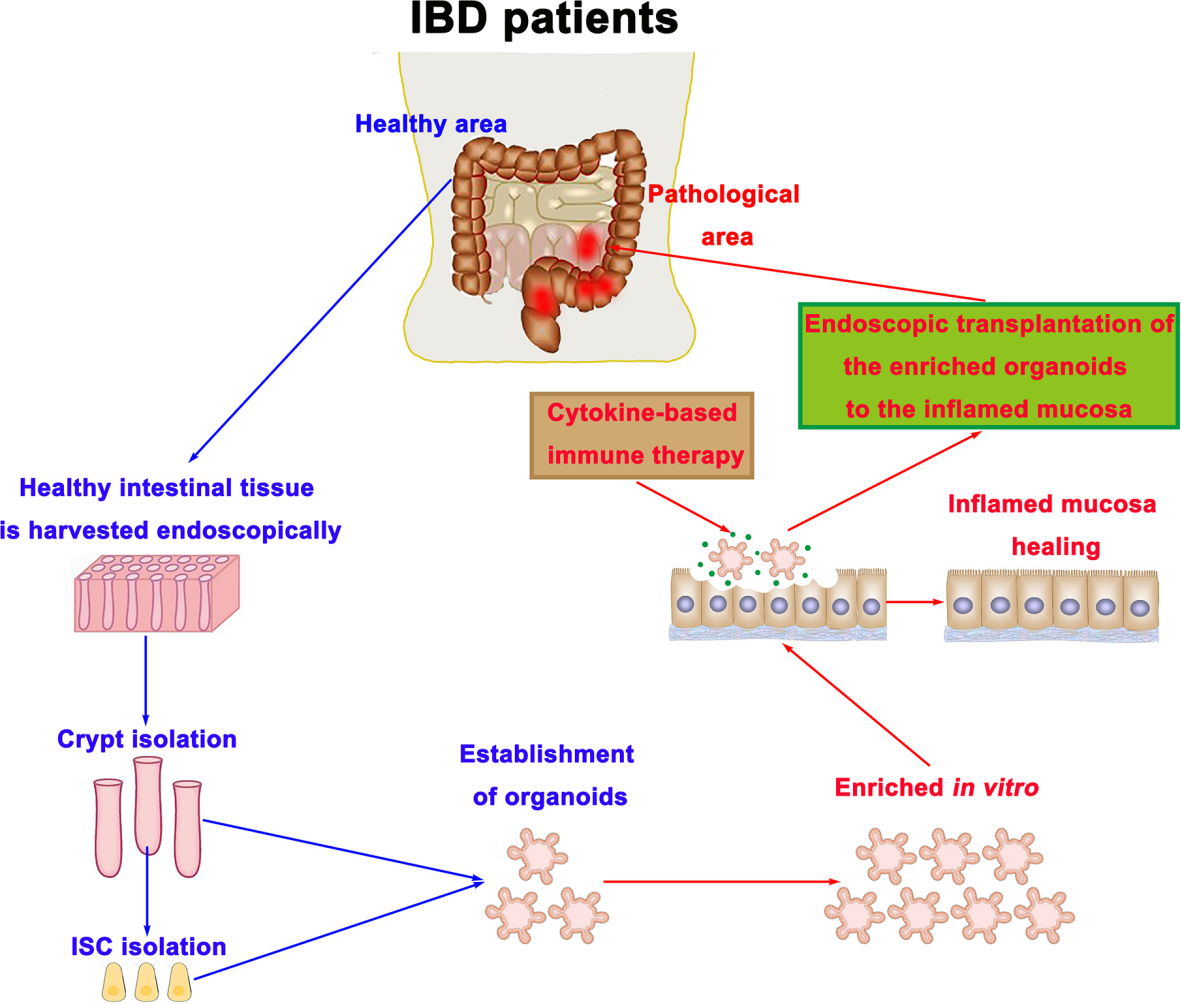
Intestinal stem cell therapy strategy in IBD. Intestinal crypts and ISCs can be harvested endoscopically from healthy intestinal mucosa in IBD patients, and then expanded *in vitro* by the established organoid culture method. After growing them to a desired number of cells, they can be transplanted onto the target site through an endoscopic delivery method. ISC, intestinal stem cell; IBD, inflammatory bowel disease.

ISC, which is responsible for intestinal epithelial regeneration, can be cultured and produced organoids *in vitro (*45). Organoids are similar to the intestinal epithelium *in vivo*, with crypt and villus domains, including various epithelial cell types derived from ISC (18). This has made the transplantation of intestinal organoids into the inflamed mucosa successful, thereby accelerating the healing of the inflamed mucosa (68, 151). Further studies showed that organoids derived from the fetal intestine or the adult small intestine are also able to engraft onto the damaged epithelium of the colon, but shows the difference in their ability to adapt to the surrounding environment through a mechanism of cell plasticity (152). A recent study also demonstrated that human intestinal organoids could accelerate the damaged mucosa healing of immunodeficient mice (153). Hence, we suppose that ISC transplantation into the inflamed mucosa provides a new therapeutic approach to reconstruct the epithelial barrier in IBD (**Figure 4**). To reduce the risk of tissue rejection, ISCs can be collected from healthy intestinal mucosa in IBD patients through the endoscopic biopsy, and then expanded *in vitro* by the established organoid culture method. Moreover, when ISC is harvested endoscopically and enriched *in vitro*, genetic mutations associated with colorectal cancer should be screened because it might increase the risk of malignant transformation in the intestine after engraftment. After growing them to a desired number of cells, they can be transplanted onto the target site through an endoscopic delivery method. Presumably, considering the links between ISC and immune cells, we predict that the integration of biological agents and ISC transplantation will revolutionize the future therapy of IBD patients.

However, further researches and several technical developments are required to enable such a treatment. The development of culture protocol aimed at maximizing ISC yield has been studied over the past decades in order to enhance successful regenerative applications (154). Prostaglandin E2 and CHIR99021 were used as case studies to promote *in vitro* proliferation and colonic stem cell expansion (155, 156). Besides, recent studies demonstrated that immune cells and cytokines secreted by these cells are tightly involved in the regulation of ISC fate, which could be applied to advance the regeneration of ISC, both *in vivo* and *in vitro*. Therefore, additional studies need to verify whether the elements of the regenerative applications of human ISC are non-toxic to tissues. Moreover, at the clinical level, we currently do not know exactly what the best index is to evaluate the clinical effect of organoid transplantation and which kind of IBD patients may most benefit from such a treatment.

A large number of clinical trials should be performed to answer these key questions in the future. Finally, on a large-scale perspective, the recombinant proteins added to the culture medium of ISC are too expensive for widespread use in the clinic. It is essential to make the protocols for culturing human ISC cost-effective.

## Conclusions and Future Perspectives

Intestinal organoids derived from ISC provide promising models to investigate the close and complex interactions between immune cells and intestinal epithelial cells, especially the ISC. Studies in the past showed that T cells and ILCs have critical effects on ISC fate and function (33–35, 55–58, 60, 61). Note that MHC molecules are expressed on the surface of Lgr5^+^ ISC, meaning that these cells may consistently contact gut-resident T cells and act as antigen-presenting cells to activate T cells. Furthermore, cytokines could directly promote or restrict ISC proliferation, differentiation, and apoptosis, which makes them critical mediators in the maintenance or destruction of the intestinal epithelial barrier. Overall, the ISC-immune cell axis provides new insight into the mechanism by which immune cells regulate ISC to preserve or restore the intestinal homeostasis. Although current studies largely expand our knowledge about the immune cell-ISC axis, we must recognize that the interaction between the ISC and immune cells could be much more complex than what we understand to date. Such studies in the future will open up a new research frontier for investigations into the biology of intestinal inflammatory diseases, such as IBD.

The key objective of IBD therapy is to heal the inflamed mucosa so as to improve its clinical symptoms, disease recurrence, and resection-free survival in patients (134–136). Unfortunately, the cross-talk among intestinal epithelial cells and immune cells complicates the maintenance and regeneration (such as mucosal healing) of the epithelial barrier. Despite the complexity of the underlying mechanisms, the successful mucosal healing from a portion of IBD patients highlights the progress made in the biological therapeutic treatment of IBD (137). For the case of patients that do not respond to biological agents, transplantation of ISC to the inflamed mucosa may be a new therapeutic approach to reconstruct the epithelial barrier in IBD. Finally, considering the links between ISC and immune cells, we predict that the integration of biological agents and ISC transplantation will revolutionize the future therapy of IBD patients.

## Author Contributions

BZ takes responsibility for the integrity of the work as a whole, from inception to the published article. QH and BZ designed the manuscript. QH wrote the initial manuscript and drew the diagrams. JH and HA wrote the manuscript. HM and BZ reviewed the manuscript. All authors contributed to the article and approved the submitted version.

## Funding

This work was supported by the National Key R&D Program of China (2017YFE0129900) and the Funding of Young Talent Supporting Program of the College of Animal Science and Technology of the China Agricultural University Education Foundation (2017DKA002).

## Conflict of Interest

The authors declare that the research was conducted in the absence of any commercial or financial relationships that could be construed as a potential conflict of interest.
